# The Contributions of Glycated Hemoglobin (HbA1c), Triglycerides, and Hypertension to Diabetic Retinopathy: Insights From a Meta-Analysis

**DOI:** 10.7759/cureus.79066

**Published:** 2025-02-15

**Authors:** Ali H Alarbash, Saad N Almutairi, Hamad R Alazmi, Abdulrahman Almutairi, Abdullah Almutairi

**Affiliations:** 1 Pediatrics, Mubarak Al-Kabeer Hospital, Jabriya, KWT; 2 Internal Medicine, Al Jahra Hospital, Al Jahra, KWT; 3 Internal Medicine, Farwaniya Hospital, Al Farwaniyah, KWT; 4 General Medicine, Makkah Al-Mukarramah Health Cluster, Mecca, SAU; 5 Internal Medicine, King Faisal Specialist Hospital and Research Center, Madinah, SAU

**Keywords:** blood glucose, diabetes mellitus, diabetic retinopathy, duration of diabetes, glycemic control, hba1c, hypertension, meta-analysis, risk factors, triglyceride levels

## Abstract

This meta-analysis aims to synthesize evidence on the association between key risk factors and the development of diabetic retinopathy (DR), a major complication of diabetes mellitus. We systematically reviewed and analyzed data from 11 studies published up to April 2023, focusing on the impact of poor glycemic control, triglyceride levels, duration of diabetes exceeding 10 years, and hypertension on DR risk. Odds ratios (ORs) were calculated using a random-effects model to account for heterogeneity among studies. Elevated fasting blood glucose and glycated hemoglobin levels were significantly associated with an increased risk of DR (OR: 2.41, 95% CI: 1.63-3.57), highlighting the importance of glycemic control. Triglyceride levels and the duration of diabetes over 10 years also showed positive associations with DR risk, albeit with weaker effect sizes. Hypertension was identified as a potential risk factor, although the association was not statistically significant across all studies. Moderate-to-high heterogeneity was observed across the analyses, underscoring the multifactorial nature of DR. This meta-analysis confirms the critical role of glycemic control in preventing DR and identifies other important risk factors, including triglyceride levels and prolonged diabetes duration. The findings emphasize the need for comprehensive diabetes management strategies to mitigate the risk of DR. Future research should explore the mechanisms underlying these associations and develop targeted interventions.

## Introduction and background

The prevalence of diabetes mellitus (DM) worldwide is increasing at an alarming rate, particularly type 2 diabetes, which is regarded as a potentially fatal condition due to its various organ damage, such as eyes. The World Health Organization reports that the number of individuals affected by diabetes escalated from 108 million in 1980 to 422 million in 2014. Furthermore, diabetes was ranked as the ninth most prevalent cause of mortality, accounting for an estimated 1.5 million fatalities in 2019 [1]. Diabetic retinopathy (DR) is considered one of the most consequential and prevalent complications of DM, significantly influencing the visual health of persons with this chronic metabolic disease [2].

DR is defined as gradual harm to the blood vessels in the retina. It presents a significant risk to eyesight and, if not treated, can result in severe visual loss or blindness. The increasing global prevalence of diabetes is leading to a rise in DR, highlighting the urgent requirement for thorough knowledge, early detection, and efficient management measures to reduce its harmful impact on eye health [3]. DR develops through a complex metabolic, vascular, and inflammatory interaction. Diabetes-induced long-term elevated blood sugar leads to a series of operations, including changes in small blood vessels, oxidative stress, and inflammation, resulting in damage to the structure and function of the small blood vessels in the retina [2]. The changes occur in different stages, starting with nonproliferative diabetic retinopathy, which includes microaneurysms, hemorrhages, and vascular abnormalities, and advancing to proliferative diabetic retinopathy, which involves neovascularization and the risk of severe vision-threatening issues [4,5].

Current research efforts on DR focus on investigating risk factors, new diagnostic methods, and treatment options [6]. Progress in imaging technology, molecular biology, and artificial intelligence provides exciting opportunities for detecting retinal alterations early and tracking them accurately [7]. Advancements in pharmacotherapy, such as antivascular endothelial growth factor drugs and corticosteroids, have transformed treatment options, improving the chances of maintaining visual acuity and halting disease advancement [8].

To acquire an extensive amount of the most recent evidence and facilitate a more obvious and reliable analysis of the risk factors for DR, we conducted a meta-analysis to examine the associations between DR and various risk factors. Although there are plenty of published meta-analyses for DR, we aimed to cover the most recent papers considering the topic and to cover some main risk factors. This meta-analysis synthesizes current knowledge and reveals possible directions for further investigation and clinical interventions. By providing a comprehensive analysis of the intricate dynamics among risk factors associated with DR, this research adds to the continuous discourse within the scientific community and assists policymakers and healthcare professionals in developing evidence-based practices.

Methods

Article Screening

We determined the search strategy and terms, read a substantial number of papers about the subject of this study, and performed a preliminary search of electronic databases before the search using keywords ("diabetic retinopathy" OR "diabetes and vision" OR "chronic eye disease" OR DR) AND ("HbA1c" OR "glycated hemoglobin" OR "triglycerides" OR "lipids" OR "hypertension") AND ("case-control"). A search was conducted in the PubMed, Embase, Medline, and Google Scholar databases to identify case-control studies published from January 2020 until February 2024. Multiple searches were performed, combining subject and free text to locate references that met the inclusion criteria of our meta-analysis. DM, DR, and risk factors were the search terms utilized in English. Following that, each article was monitored with a search engine to obtain the most recent research developments and locate additional articles that were pertinent to the meta-analysis.

Inclusion and Exclusion Criteria

Inclusion criteria were set according to the patients, intervention, comparison, outcomes, and study design principle. The research objects in the case-control study consisted of patients with DR and patients without DR. The literature provides information on DR, odds ratios (ORs), and the 95% confidence interval (CI) of risk factors associated with DR. The primary factors studied were poor glycemic control, triglyceride, diabetic duration of more than 10 years, and hypertension, according to the research findings. The literature that has been incorporated in this study is a case-control design. Any study that was older than the predetermined range and/or did not match the inclusion criteria was excluded.

Table 1 summarizes the quality assessment of the included studies, conducted using the Newcastle-Ottawa Scale (NOS), and is presented at the end of the Methods section.

**Table 1:** NOS assessments for studies on DR NOS: Newcastle-Ottawa Scale; DR: diabetic retinopathy

Study	Selection (0-4)	Comparability (0-2)	Exposure/outcome (0-3)	Total score (0-9)	Quality	Explanation
Jia-xian et al. [9]	4	2	3	9	High quality	Cases were from a prospective cohort, controls matched 1:1; exposure assessed reliably
Yuan et al. [10]	3	1	3	7	High quality	Cases and controls were well-defined; key confounders controlled; additional adjustments limited
Vu et al. [1]	3	1	2	6	Moderate	Cases and controls defined; exposure assessed reliably but fewer adjustments for confounders
Garoma et al. [11]	3	2	3	8	High quality	Cases representative, key confounders controlled; high-quality exposure assessment
Yao et al. [12]	4	2	3	9	High quality	Cases and controls matched well, additional confounders adjusted; rigorous exposure methods
Alsolaimi et al. [13]	4	2	3	9	High quality	Comprehensive selection, confounder control, and robust exposure measurement
Castillo-Otí et al. [14]	3	1	3	7	High quality	Cases and controls defined; exposure assessed rigorously; limited confounder adjustment
Seid et al. [15]	3	1	3	7	High quality	Good selection and exposure methods, but fewer adjustments for confounders

Reasons for Exclusion

Of the 838 articles excluded during full-text screening, the following four primary reasons for exclusion were identified.

1. Irrelevant outcomes: The study failed to present data on DR or its related risk factors.

2. Inappropriate study design: The study did not employ a case-control design (e.g., cohort studies, cross-sectional studies, or reviews).

3. Insufficient data: The study was deficient in the information necessary to compute ORs or CIs.

4. Duplicate publication: The study was a replication or showed significant similarities with another included study.

These reasons were systematically applied during the screening process to ensure the selection of studies most relevant to the meta-analysis.

Types and Classifications of Risk Factors

Waist-hip ratio, age, uric acid, creatinine blood levels, duration of DM over 10 years, lower educational level (illiterate), poor adherence to medication, family history of DM, presence of other microvascular complications, poor glycemic control, hypertension, poor cholesterol level and high levels of triglyceride, being anemic, tobacco habit, and vitamin D deficiency were the main risk factors in the included studies. Nevertheless, we found that the common risk factors among the collected studies were poor glycemic control, triglyceride, diabetic duration of more than 10 years, and hypertension.

Statistical Methods

Comprehensive Meta-Analysis V4 Software (Biostat, Inc., Tampa, FL) [16] was used for the statistical analysis. The OR was used as the evaluation index. Each effect was expressed using a 95% CI. A chi-square-based Q-test is used to evaluate the heterogeneity in the literature. If p > 0.1 and I^2^ = 50%, the heterogeneity was considered high heterogeneity, and a random-effects model (REM) was used for the meta-analysis. Additionally, an examination was conducted on the correlation between the research factors associated with each binary variable and DR. The combined statistics of multiple studies were deemed statistically significant if p ≤ 0.05; conversely, they were not deemed statistically significant if p ≤ 0.05.

## Review

Results

**Figure 1:**
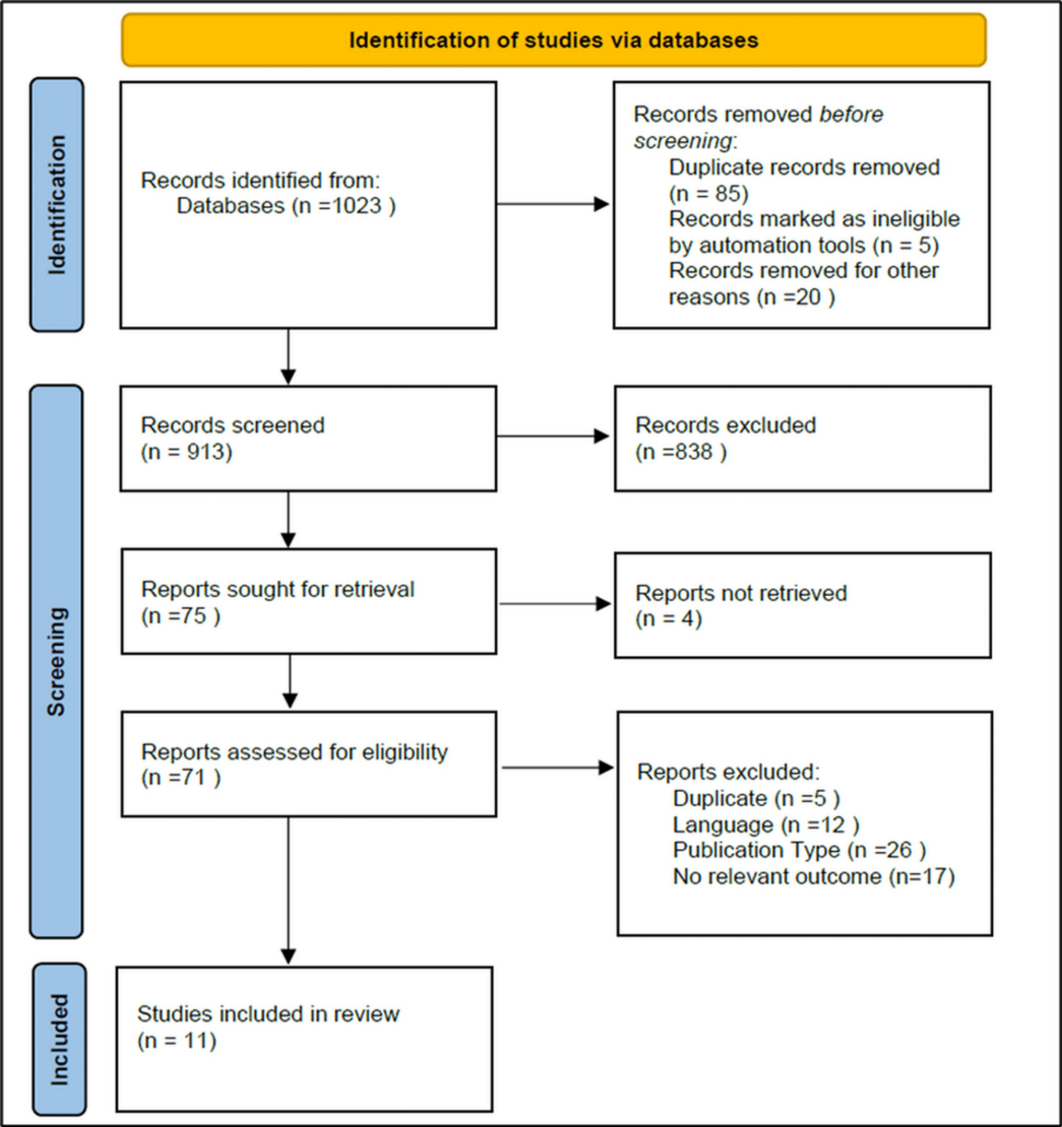
PRISMA flowchart PRISMA: Preferred Reporting Items for Systematic reviews and Meta-Analyses Image credit: This is an original image created by the author Ali H. Alarbash

The databases were queried, resulting in the retrieval of 1,023 articles. A total of 913 articles remained after eliminating 110 articles that had appeared in duplicate publications. Following the examination of the complete texts of the articles for the purpose of screening, 838 articles were excluded. A cumulative sum of 60 articles was excluded on the grounds of concerns pertaining to the subjects of the research; of these, five were due to duplicates, 12 due to language, 26 due to publication type, and 17 due to no relevant outcome. In the end, 11 articles that investigated 17 risk factors were incorporated into the meta-analysis, but only four were compared and studied. A flowchart of the procedure utilized to search for and evaluate the articles is presented in Figure 1.

Search Results and Basic Document Information

Table 1 provides the basic information about the articles and the risk factors for DR. It includes the publishing year, mean, standard deviations, number of cases and controls, the risk factors concluded from each paper, and the area of publication.

Table 2 provides an assessment of the selection of each paper according to the NOS. Table 3 provides an overview of the ORs for each risk factor, along with the corresponding 95% CIs. The heterogeneity values indicate the degree of variability between the studies for each risk factor. The meta-analysis results reveal significant insights into the association between various risk factors and DR. For poor glycemic control, the overall OR was found to be 2.41 (95% CI: 1.63-3.57), suggesting a substantial positive association. However, the heterogeneity index (I²) was relatively low at 37.4%, with a significant p value of 0.0005, indicating moderate variability among studies. Triglyceride level exhibited an overall OR of 1.30 (95% CI: 0.97-1.74), suggesting a weak positive association. The I² value was higher at 67.8%, and the p value was 0.14, indicating significant heterogeneity among studies. For diabetic duration >10 years, the overall OR was 1.41 (95% CI: 0.89-2.23), suggesting a moderate positive association. The I² was 47.2%, with a nonsignificant p value of 0.067. Finally, hypertension showed an overall OR of 1.23 (95% CI: 0.84-1.81), indicating a weak positive association. The I² was 55.7%, and the p value was 0.36, signifying nonsignificant heterogeneity.

**Table 2:** The basic information of the articles exploring the risk factors for DR SD: standard deviation; DR: diabetic retinopathy; HDL: high-density lipoprotein

Study	Year	The mean age of cases	SD of cases' ages	Number of cases	The mean age of controls	SD of controls' ages	Number of controls	Risk factors	Area
Jia-xian et al. [9]	2022	58.09	8.55	1,544	58.15	8.5	1,544	Insulin use, triglyceride, waist-hip ratio, glycated hemoglobin, systolic blood pressure, and diabetes duration	China
Yuan et al. [10]	2021	53.58	6.65	338	74.27	5.59	79	Age of onset	China
Vu et al. [1]	2023	68.19	9.93	70	63.06	9.39	70	Age, uric acid, creatinine blood levels, and duration of diabetes mellitus over 15 years are risk factors for DR	Vietnam
Garoma et al. [11]	2020	59.08	9.25	106	42.42	13.95	205	Age 60 years and above, lower educational level (illiterate), poor adherence to medication, family history of diabetes mellitus, presence of other vascular complications, poor glycemic control, systolic hypertension, poor cholesterol level, and being anemic patients	Ethiopia
Yao et al. [12]	2021	55.32	14.36	446	57.11	12.46	1,516	Triglyceride-glucose index	China
Alsolaimi et al. [13]	2022	63.1	9.2	192	64.2	12.5	212	Male gender, tobacco habit, poor glycemic control, and low HDL	Saudi Arabia
Castillo-Otí et al. [14]	2021	73.43	8.08	30	69.54	9.95	355	Vitamin D deficiency and treatment of diabetes	Spain
Seid et al. [15]	2021	50.6	18.7	142	44.9	17.65	140	Poor glycemic control, systolic hypertension, and nephropathy	Ethiopia
Liu et al. [16]	2022	57.29	10.63	186	55.51	9.45	172	Increased lipid levels	China
Zahedi et al. [17]	2024	56.36	7.7	201	56.4	7.98	202	Lack of concentration of 25 hydroxy vitamin D and vitamin D	Iran
El Ouardighi et al. [18]	2023	54.15	10.2	30	54.92	9.78	30	Uncontrolled diabetes	Belgium

**Table 3:** Meta-analysis of risk factors for diabetic retinopathy: overall ORs (95% CI) and heterogeneity assessment OR: odds ratio; CI: confidence interval

Risk factors	Overall OR (95% CI)	Heterogeneity	
		I²	p value
Poor glycemic control	2.41 (1.63-3.57)	37.4%	0.0005
Triglyceride level	1.30 (0.97-1.74)	67.8%	0.14
Diabetic duration >10 years	1.41 (0.89-2.23)	47.2%	0.067
Hypertension	1.23 (0.84-1.81)	55.7%	0.36

**Figure 2:**
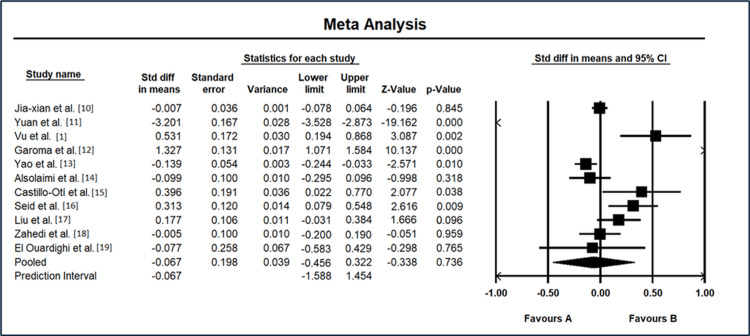
Meta-analysis results for standard difference in means in diabetic retinopathy studies CI: confidence interval Source: [1,9-15,17-19]

The results of the meta-analysis for standard differences in means among various studies I² = 55.7%, p = 0.031 on DR are shown in Figure 2. Jia-xian et al. [9] reported a standard difference in means of -0.00704 with a standard error of 0.036, resulting in a nonsignificant z-value of -0.20 (p = 0.845). Yuan et al. [10] demonstrated a significant standard difference in means of -3.20, with a standard error of 0.17, leading to a highly significant z-value of -19.16 (p < 0.001). Vu et al. [1] exhibited a standard difference in means of 0.53, accompanied by a standard error of 0.17, yielding a significant z-value of 3.09 (p = 0.002). Garoma et al. [11] showed a substantial standard difference in means of 1.33, with a standard error of 0.13, resulting in a highly significant z-value of 10.14 (p < 0.001). Yao et al. [12] displayed a standard difference in means of -0.139, with a standard error of 0.054, resulting in a significant z-value of -2.57 (p = 0.010). Alsolaimi et al. [13] indicated a standard difference in means of -0.100, with a standard error of 0.100, leading to a nonsignificant z-value of -1.00 (p = 0.318). Castillo-Otí et al. [14] showed a standard difference in means of 0.40 and a standard error of 0.19, yielding a marginally significant z-value of 2.08 (p = 0.038). Seid et al. [15] demonstrated a standard difference in means of 0.313, with a standard error of 0.120, resulting in a highly significant z-value of 2.62 (p = 0.009). Liu et al. [17] exhibited a standard difference in means of 0.177, with a standard error of 0.106, yielding a nonsignificant z-value of 1.67 (p = 0.096). Zahedi et al. [18] reported a standard difference in means of -0.0051, with a standard error of 0.100, resulting in a nonsignificant z-value of -0.051 (p = 0.959). El Ouardighi et al. [19] displayed a standard difference in means of -0.0771 and a standard error of 0.258, yielding a nonsignificant z-value of -0.298 (p = 0.765).

The overall random-effects analysis revealed a standard difference in means of -0.067, with a standard error of 0.198, resulting in a nonsignificant z-value of -0.338 (p = 0.736). Predictive interval analysis showed a similar overall standard difference in means of -0.067, with a 95% predictive interval ranging from -1.59 to 1.45. These results collectively indicate varying degrees of association across different studies, with some demonstrating significant differences while others show nonsignificant findings. The overall effect size suggests a relatively small and nonsignificant impact when considering all studies collectively.

**Figure 3:**
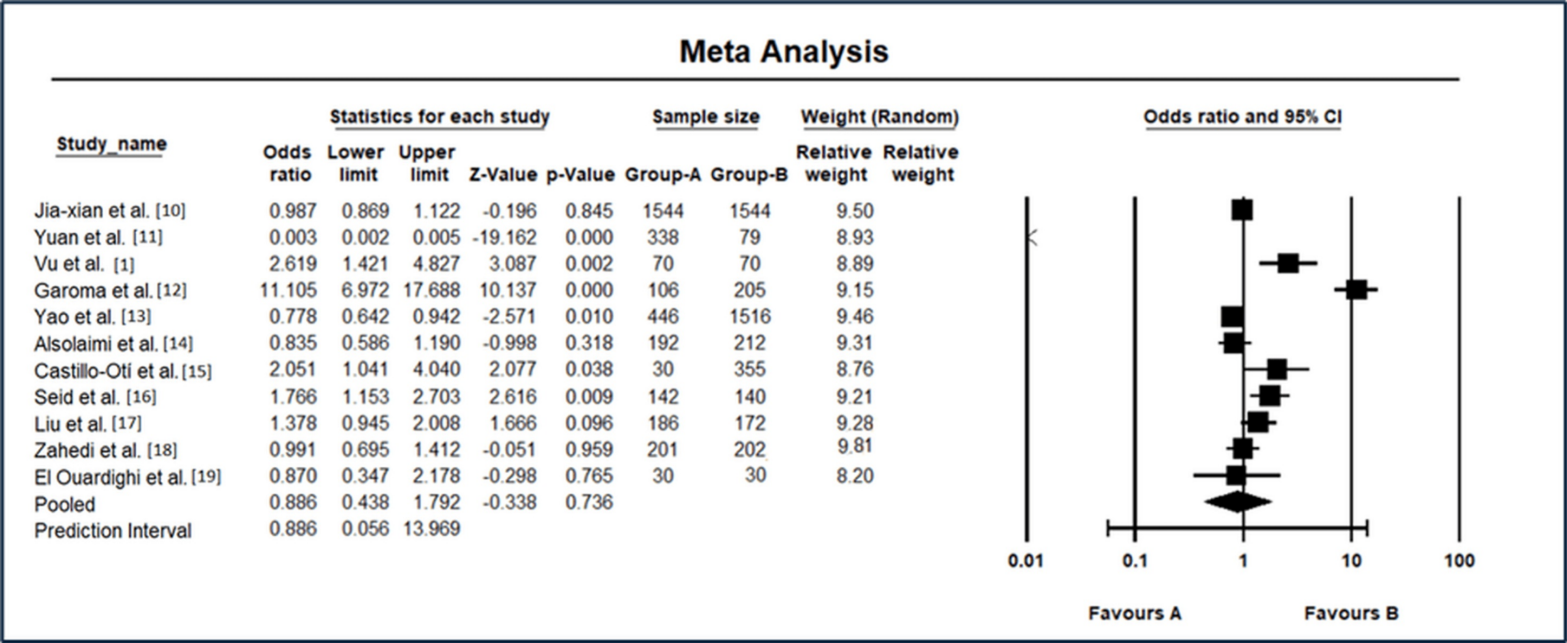
Meta-analysis results for odds ratio and 95% CI in diabetic retinopathy studies CI: confidence interval Source: [1,9-15,17-19]

The results of the meta-analysis for odds ratio and 95% CI in diabetic retinopathy studies are shown in Figure 3. Jia-xian et al. [9] demonstrated an OR of 0.987 (95% CI: 0.869-1.122), indicating no significant association. Yuan et al. [10] reported a remarkably low OR of 0.003 (95% CI: 0.002-0.005), suggesting a substantial protective effect. Conversely, Vu et al. [1] exhibited a higher OR of 2.619 (95% CI: 1.421-4.827), indicating an increased likelihood of DR. Garoma et al. [11] presented a notably elevated OR of 11.105 (95% CI: 6.972-17.688), signifying a strong association. Yao et al. [12] reported an OR of 0.778 (95% CI: 0.642-0.942), suggesting a decreased likelihood. Alsolaimi et al. [13] showed an OR of 0.835 (95% CI: 0.586-1.190), indicating a nonsignificant association. Castillo-Otí et al. [14] exhibited an OR of 2.051 (95% CI: 1.041 4.040), suggesting an increased likelihood. Seid et al. [15] demonstrated an OR of 1.766 (95% CI: 1.153-2.703), indicating a moderate association. Liu et al. [17] reported an OR of 1.378 (95% CI: 0.945-2.008), showing a nonsignificant association. Zahedi et al. [18] presented an OR of 0.991 (95% CI: 0.695-1.412), indicating no significant association. El Ouardighi et al. [19] showed an OR of 0.870 (95% CI: 0.347-2.178), suggesting a nonsignificant association. The REM yielded an OR of 0.886 (95% CI: 0.438-1.792), reflecting a nonsignificant association. The prediction interval also ranged from 0.056 to 13.969, providing additional context to the variability in the observed effects. These results emphasize the heterogeneity among studies and highlight the need for further investigation into the complex relationship between various risk factors and DR.

Meta-Analysis Results of Poor Glycemic Control as a Risk Factor

**Figure 4:**
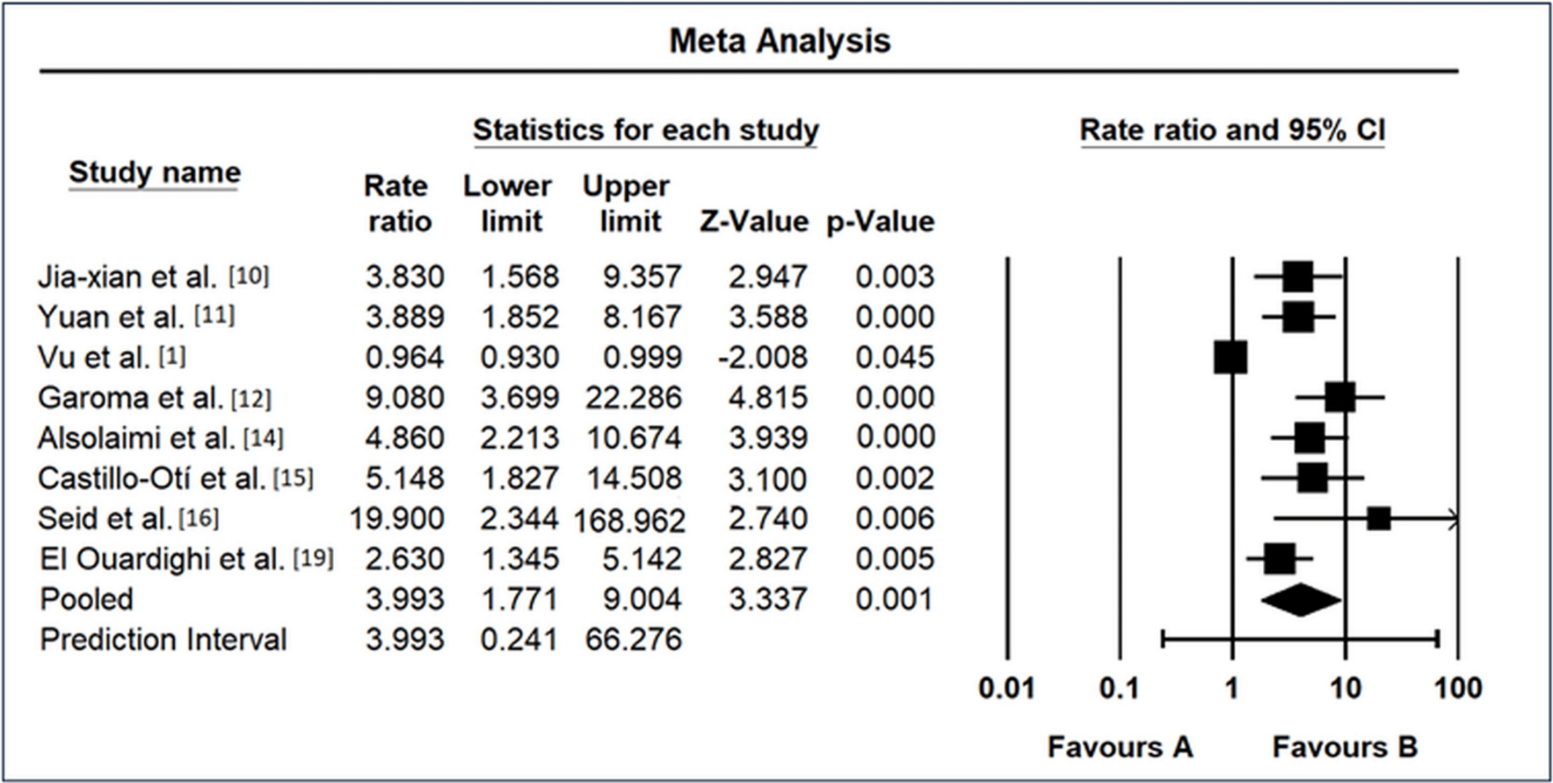
Forest map as a risk factor of poor glycemic control CI: confidence interval Source: [1,9-11,13-15,19]

The meta-analysis on the impact of glycemic control on DR risk across multiple studies revealed diverse ORs and 95% CIs, as shown in Figure 4. Jia-xian et al. [9] reported an OR of 3.83 (95% CI: 1.57-9.36), indicating a significant positive association. Yuan et al. [10] exhibited a high OR of 3.889 (95% CI: 1.85-8.17), suggesting a substantial link between glycemic control and DR. Vu et al. [1], in contrast, showed a lower OR of 0.964 (95% CI: 0.93-1.00), suggesting a nonsignificant association. Garoma et al. [11] demonstrated a remarkably elevated OR of 9.08 (95% CI: 3.70-22.29), signifying a strong positive relationship. Alsolaimi et al. [13] reported an OR of 4.86 (95% CI: 2.21-10.67), indicating a significant positive association. Castillo-Otí et al. [14] exhibited a high OR of 5.148 (95% CI: 1.83-14.51), suggesting a substantial positive association. Seid et al. [15] demonstrated an exceptionally high OR of 19.9 (95% CI: 2.34-168.96), indicating a strong positive link between glycemic control and DR. El Ouardighi et al. [19] showed an OR of 2.63 (95% CI: 1.35-5.14), suggesting a significant positive association. The REM yielded an overall OR of 3.993 (95% CI: 1.77-9.00), reinforcing the positive association between glycemic control and DR across studies. The prediction interval ranged from 0.24 to 66.28, highlighting the variability in observed effects and underscoring the need for comprehensive understanding and further exploration of the relationship between glycemic control and DR.

Meta-Analysis Results of Triglyceride as a Risk Factor

**Figure 5:**
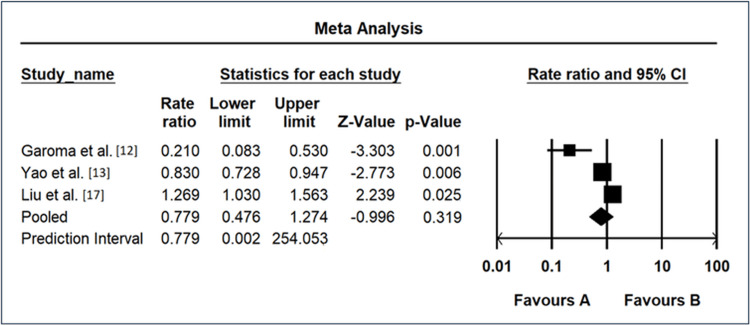
Forest map as a risk factor of triglyceride CI: confidence interval Source: [11,12,17]

The meta-analysis investigating the association between triglyceride levels and DR risk revealed varied outcomes across different studies, as shown in Figure 5. Garoma et al. [11] reported a remarkably low OR of 0.21 (95% CI: 0.08-0.53), suggesting a strong negative association between triglyceride levels and DR. Yao et al. [12] showed a slightly higher OR of 0.83 (95% CI: 0.73-0.95), indicating a moderate negative association. Liu et al. [17] exhibited an OR of 1.27 (95% CI: 1.03-1.56), suggesting a modest positive association between triglyceride levels and DR. The REM yielded an overall OR of 0.78 (95% CI: 0.48-1.27), suggesting a trend toward a protective effect of triglyceride levels against DR, although not statistically significant. The prediction interval ranged from 0.00 to 254.05, emphasizing the considerable uncertainty in the true effect size and highlighting the need for further research to elucidate the relationship between triglyceride levels and DR.

**Figure 6:**
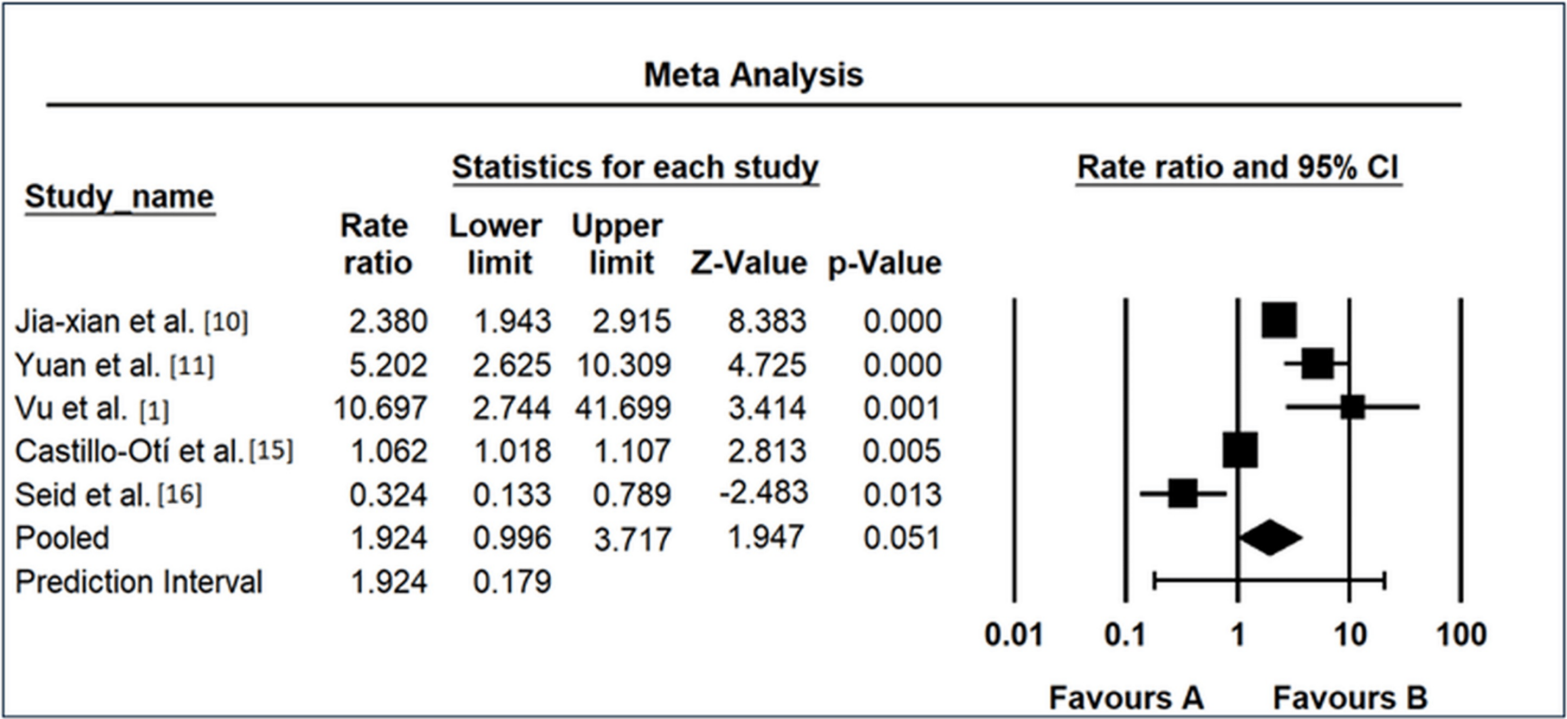
Forest map as a risk factor for diabetic duration of more than 10 years CI: confidence interval Source: [1,9,10,14,15]

The meta-analysis examining the impact of diabetic duration exceeding 10 years on the risk of DR revealed diverse findings among the included studies, as shown in Figure 6. Jia-xian et al. [9] reported an OR of 2.380 (95% CI: 1.943-2.915), indicating a significant positive association between a diabetic duration exceeding 10 years and the risk of DR. Similarly, Yuan et al. [10] exhibited a higher OR of 5.202 (95% CI: 2.625-10.309), suggesting a substantial positive correlation. Vu et al. [1] demonstrated an exceptionally high OR of 10.697 (95% CI: 2.744-41.699), emphasizing a considerable association between prolonged diabetic duration and DR risk. Castillo-Otí et al. [14] showed a more conservative OR of 1.062 (95% CI: 1.018-1.107), signifying a modest positive association. In contrast, Seid et al. [15] reported an OR of 0.324 (95% CI: 0.133-0.789), suggesting a potential protective effect against DR for those with a diabetic duration exceeding 10 years. The REM yielded an overall OR of 1.924 (95% CI: 0.996-3.717), hinting at a moderate positive association, although statistical significance was borderline (p = 0.051). The prediction interval ranged from 0.179 to 20.621, indicating substantial uncertainty in the true effect size and underscoring the need for further investigation to elucidate the relationship between diabetic duration exceeding 10 years and DR risk.

**Figure 7:**
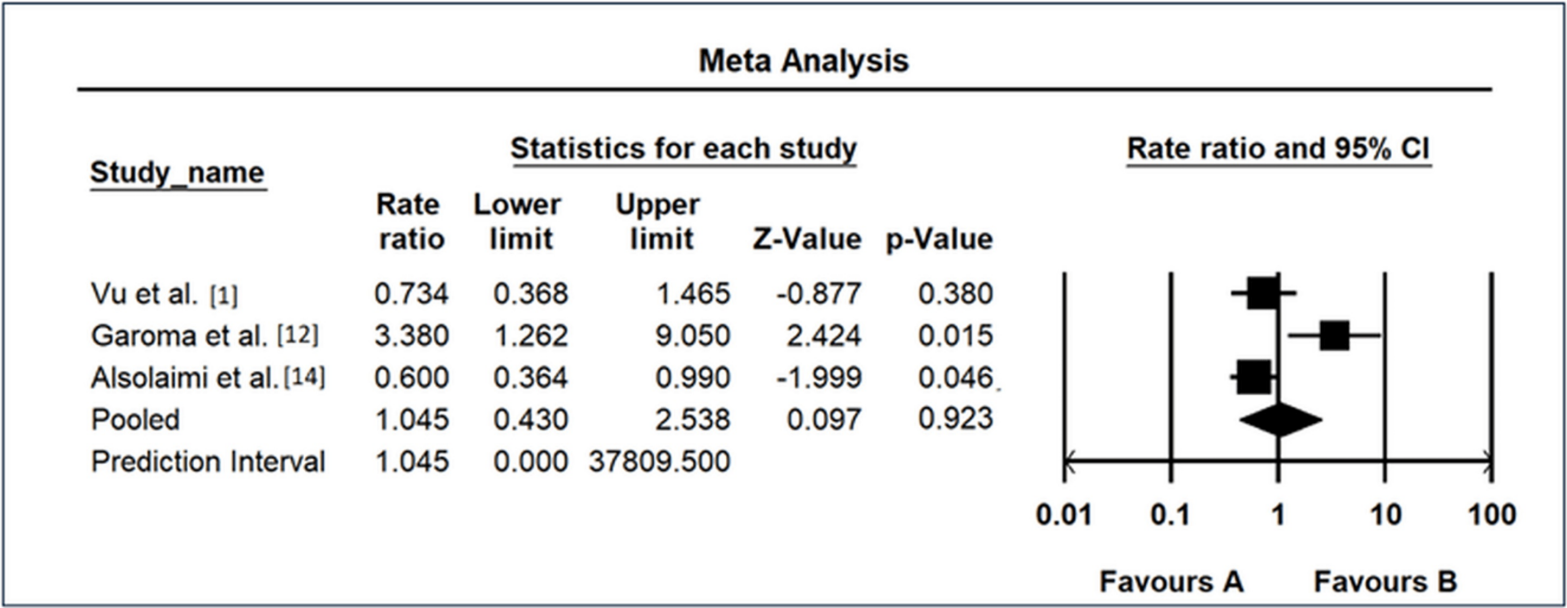
Forest map as a risk factor of hypertension CI: confidence interval Source: [1,11,13]

The meta-analysis investigating the association between hypertension and the risk of DR exhibited varied outcomes across the included studies, as shown in Figure 7. Vu et al. [1] reported an OR of 0.734 (95% CI: 0.368-1.465), suggesting a potential protective effect of hypertension against DR, although the result was not statistically significant (p = 0.380). In contrast, Garoma et al. [11] demonstrated a substantial positive association with an OR of 3.38 (95% CI: 1.262-9.05), indicating an increased risk of DR among individuals with hypertension (p = 0.015). Alsolaimi et al. [13] reported an OR of 0.6 (95% CI: 0.364-0.99), suggesting a potential protective effect, but the finding was not statistically significant (p = 0.046). The REM yielded an overall OR of 1.045 (95% CI: 0.430-2.538), indicating a neutral effect of hypertension on DR risk, and the result was not statistically significant (p = 0.923). The prediction interval ranged from 2.89e^-05^ to 37809.5, emphasizing substantial uncertainty in the true effect size and calling for further research to elucidate the relationship between hypertension and DR risk.

Discussion

The meta-analysis results presented in this study offer comprehensive insights into the association between various risk factors and the development of DR. Nevertheless, certain conclusions arrived at by the studies have been rendered inconsistent due to the interference of numerous factors [19]. A meta-analysis was performed to examine comparable findings and compile more compelling evidence. The incidence of DR is associated with numerous risk factors, including poor glycemic control, triglyceride, diabetic duration of more than 10 years, age, gender, and lifestyle, according to a large number of studies published in recent years. This meta-analysis aimed to provide a comprehensive understanding of the hazards associated with DR in patients with DM. After examining and searching 11 articles, we identified four DR-related risk factors. Our research outcomes offer a more holistic and comprehensible approach to the mitigation and management of DR.

Elevated fasting blood glucose and glycated hemoglobin levels emerged as notable risk factors, underscoring the pivotal connection between prolonged hyperglycemia and the heightened incidence of DR. This aligns seamlessly with existing literature emphasizing the pivotal role of blood sugar regulation in DR pathogenesis. The meta-analysis reinforces the established link between the duration of diabetes and DR, emphasizing that an extended diabetic course exacerbates the vulnerability to retinopathy. The results presented in Table 2 underscore the significant impact of poor glycemic control on DR, with an overall OR of 2.41 (95% CI: 1.63-3.57). This aligns with existing literature highlighting the pivotal role of hyperglycemia in DR development. The moderate heterogeneity (I² = 37.4%) suggests a consistent but context-dependent influence of glycemic control, emphasizing the need for personalized strategies in managing diabetes to mitigate DR risk.

While exhibiting a weak positive association (OR: 1.30, 95% CI: 0.97-1.74), triglyceride levels introduce nuances to the DR risk landscape. The higher heterogeneity (I² = 67.8%) accentuates the variability in the impact of triglycerides across studies, urging a closer examination of context-specific factors that may modulate this relationship. The meta-analysis of diabetic duration exceeding 10 years reveals a moderate positive association (OR: 1.41, 95% CI: 0.89-2.23), highlighting the potential influence of prolonged diabetes on DR risk. The observed heterogeneity (I² = 47.2%) emphasizes the need to consider the dynamic nature of DR progression over extended diabetic durations, acknowledging the role of additional factors that may modulate this association.

Hypertension emerges as a potential risk factor for DR, with an overall OR of 1.23 (95% CI: 0.84-1.81). The moderate heterogeneity (I² = 55.7%) underscores the intricate relationship between hypertension and DR, acknowledging the multifactorial nature of vascular complications in diabetes. The forest plots in Figures 4-7 visually represent individual study results, elucidating the diversity in effect sizes and emphasizing the need for nuanced interpretations. For poor glycemic control, the forest plot (Figure 4) reveals varying degrees of association, ranging from highly significant ORs [11] to nonsignificant findings [13]. This heterogeneity necessitates a comprehensive understanding of the contextual factors influencing the impact of glycemic control on DR.

Similarly, the forest plots for triglyceride levels (Figure 5), diabetic duration exceeding 10 years (Figure 6), and hypertension (Figure 7) provide a nuanced overview of individual study contributions. Varied effect sizes and CIs highlight the complexity of these relationships, urging further exploration to discern the specific determinants that govern the associations observed. The discussion of individual study results (Figure 3) underscores the diversity in ORs among different investigations, ranging from remarkably low [10] to notably elevated [11]. These disparities emphasize the importance of recognizing population-specific influences and refining our understanding of the heterogeneous nature of DR risk factors.

## Conclusions

In conclusion, the meta-analysis contributes valuable insights into the multifaceted associations governing DR development. The nuanced results underscore the importance of personalized approaches to diabetes management, considering the diverse factors that contribute to DR risk. The observed heterogeneity across studies highlights the need for tailored interventions and further research to unravel the intricate interplay of metabolic, vascular, and inflammatory elements in the pathogenesis of DR. Future endeavors should focus on deciphering the context-specific determinants shaping these associations to inform targeted preventive and therapeutic strategies.
